# Normal Sweat Secretion Despite Impaired Growth Hormone-Insulin-Like Growth Factor-I Axis in Obese Subjects

**DOI:** 10.1155/2011/493840

**Published:** 2011-08-04

**Authors:** Michael Højby Rasmussen, Anders Juul, Katharina M. Main, Jannik Hilsted

**Affiliations:** ^1^Department of Endocrinology, Hvidovre Hospital, University of Copenhagen, 2650 Copenhagen, Denmark; ^2^Department of Growth and Reproduction, Rigshospitalet, University of Copenhagen, 2100 Copenhagen, Denmark

## Abstract

Adults with GH deficiency are known to exhibit reduced sweating. Whether sweating capacity is impacted in obese subjects with impaired GH secretion have not previously been investigated. The main objective was to investigate sweat secretion rate and the GH-IGF-I axis in obese subjects before and after weight loss. Sixteen severely obese women (BMI, 40.6 ± 1.1 kg/m^2^) were investigated before and after a diet-induced weight loss. Sixteen age-matched nonobese women served as controls. The obese subjects presented the characteristic decreased GH release, hyperinsulinaemia, increased FFA levels, and impaired insulin sensitivity, which all were normalised after diet-induced weight loss of 30 ± 5 kg. Sweat secretion rates were similar comparing obese and nonobese subjects (78 ± 10 versus 82 ± 9 mg/30 minutes) and sweat secretion did not change after a diet-induced weight loss in obese subjects. We conclude that although obese subjects have markedly reduced GH release and impaired IGF-I levels, sweat secretion rate is found to be normal.

## 1. Introduction

Sweating capacity as measured by sweat secretion rates has been reported reduced in adult patients with GH deficiency [1–4] as well as in children with GH deficiency (GHD) and GH insensitivity syndromes [5, 6]. Furthermore, sweat secretion has been found correlated to circulating IGF-I levels [1, 4, 7], and GH deficiency have been reported to have a profound effect on thermoregulation [2, 8]. These observations in patients with GHD suggest that GH have a role in thermal regulation, via an effect on sweating capacity, and the reduced sweating is part of the abnormalities observed in patients with GHD [7, 9], as well as that some of the positive effects reported following GH replacement therapy might be due to a normalization of sweat secretion [7]. Normal sweating is of importance for maintaining correct temperature regulation and if disturbed can cause significant discomfort. The mechanism of GH action on sweating is not clear; however, GH receptors have been demonstrated on sweat glands, and GH may function as a trophic factor for sweat glands [10]. It has been suggested that GH hyper- and hyposecretory states result in changes in the growth and metabolic status of sweat glands, with alterations in sweat gland innervation [4] and a reduced number of sweat glands has been reported in adults with GHD [11]. 

Adults with GHD are characterised by perturbations in body composition, lipid metabolism, and cardiovascular risk profile [12]. It is well established that adult GHD usually is accompanied by an increase in fat accumulation and these changes in body composition are associated with metabolic derangements including insulin resistance [12]. Obese subjects as adults with GHD have multiple endocrine abnormalities including insulin resistance, severely suppressed spontaneous and stimulated GH release [13–15], and impaired circulating IGF-I levels [13]. The obesity-associated decrease in GH secretion is partially or fully recovered after diet-induced or surgical-induced reduction of fat mass [13, 14]. In addition, sweat gland activity is reported impaired in obese subjects [16], and heat-waves can cause major discomfort to obese subjects. Although, the many similarities between adults with GHD and obese subjects are well recognised, there are no published data available on sweating capacity in obese subjects and the relationship to the GH-IGF-I axis. Thus, the primary aim of the study was to investigate sweat secretion rate in obese subjects as well as in relation to the GH-IGF-I axis and the impact of a diet-induced weight loss on sweating. Secondary objective was to relate sweat secretion and GH-IGF-I to endocrine abnormalities including hyperinsulinaemia, and elevated FFA often observed both in obese subjects and adults with GHD.

## 2. Methods

### 2.1. Subjects

We studied 16 obese female subjects, with a mean age of 29.5 ± 1.4 years and with a mean BMI of 40.6 ± 1.1 kg/m²; these were morbidly obese subjects, who underwent a diet-induced weight loss program. At the conclusion of the weight-loss program, 8 obese women completed the weight loss program with a weight-loss of approximately 30% of their initial body weight corresponding to a weight-loss of 30 ± 5 kg and a mean body mass index (BMI) of 27.0 ± 1.4 kg/m². The remaining 8 obese women dropped out during the study due to lack of motivation. The control group consisted of 16 normal lean female subjects with a mean age of 27.0 ± 1.1 years and a mean BMI of 23.2 ± 0.4 kg/m². All the subjects were nondiabetic and premenopausal. All the subjects gave informed consent. The revised Helsinki 2 Declaration was observed, and the study was approved by the Copenhagen Municipal Ethical Committee.

### 2.2. Weight-Loss Study

The obese subjects participated in a structured, outpatient weight-loss program where the goal was to achieve ideal body weight. During the initial phase the subjects consumed a commercial very-low-calorie diet that provided 1.6 megajoule (MJ)/day. The patients visited the outpatient clinic weekly for body weight measurements and nutrition counselling. When body weight had decreased to approximately 130% of ideal body weight, the patients were instructed to discontinue the 1.6 MJ/day diet and begin consuming 5.0 MJ/day diet of normal food items. During this period, the patients used the diet and nutrition materials that had been supplied to them and discussed during the counselling sessions. When ideal body weight was achieved or when no further weight could be lost, the patients were instructed to begin consuming a basic 8.0 MJ/d diet (15% protein, 55% carbohydrate, and 30% fat) which was individually adjusted for each patient to obtain energy balance. Energy intake was further adjusted down or up for each subject on a weekly basis (during weekly dietary counselling) to reach a final, stable body weight (e.g., if body weight began to increase, daily energy intake was reduced). Sampling in the postobese patients was performed after body weight had remained stable (±1.5 kg) for ≥1 month after switching to the last diet.

### 2.3. Twenty-Four-Hour Study

For each 24-h sampling period the subjects were admitted to the metabolic ward at 07:30 AM. after an overnight fast. A cannula (Viggo AB, Vingmed, Denmark) was placed in a forearm vein. Blood withdrawal commenced 30 min after venipuncture through a nonthrombogenic catheter (Carmeda; Viggo AB, Vingmed, Denmark) inserted through the cannula and connected to an automatic constant withdrawal pump (withdrawal rate was 3.5 mL/h). Collection tubes were changed at 20-min intervals over the following 24-h period giving a series of 72 sequential samples for each subject. The technique allows the participants to sleep during the night and to move freely around during the day. For GH measurements all 72 serum samples were assayed (total *n* = 5760). The basal blood samples were taken after the subjects had fasted overnight.

### 2.4. Analyses

Evaluation of sweat secretion was performed as previously described [17]. In the resting subject, sweating was induced on the flexor side of the distal forearm by iontophoresis with 0.2% pilocarpine chloride, using a current of 2 mA for 5 minutes. Two filter papers (Whatman Ashless Roundfilters, diameter 2 cm) were soaked in pilocarpine solution and were positioned underneath the electrode. Two quadrangular negative electrodes (size 3 × 3 cm) were placed on the flexor side of the upper arm. After 5 minutes, the area of iontophoresis was rinsed with deionized water and 61% ethanol and dried thoroughly. Sweat was collected for a 30 minute-period with the three filter papers sealed with roundel of plastic tape to prevent evaporation. Sweat mass was measured by weighing filter paper (Mettler Scales, precision ±0.1 mg) before and after collection. 

Serum GH concentration was determined using a time-resolved immunofluorometric assay (TR-IFMA, PerkinElmer LifeSciences, Türku, Finland). IGF-I was determined in acid-ethanol-extracted serum by a specific radioimmunoassay [18]. Insulin was determined using an enzyme-linked two-site immunoassay as described by Andersen et al. [19]. FFA concentrations were determined by a previously described microfluorometric method [20]. 

### 2.5. Calculations

The area under the curve (AUC) of 24-hour GH release was estimated above the zero level as previously described [21]. The Quantitative Insulin Sensitivity Check Index (QUICKI) was calculated from the fasting insulin and glucose values; QUICKI = 1/[log  insulin + log  glucose] as described by Katz et al. [22]. Body mass index was calculated as weight (kg)/height (m²). Body weight was measured to the nearest 0.1 kg with an electronic scale (Seca 070; Seca, Copenhagen). Twenty-four-hour GH release and IGF-I data have previously been reported separately [23].

### 2.6. Statistics

Mann-Whitney rank sum test was used when comparing data between different groups (unpaired data). Wilcoxon signed rank test was applied when comparing paired data from the previously obese subjects to the same eight subjects in the prediet obese state. Linear regression analysis was performed to identify independent effects of hormonal factors (FFA, insulin, and IGF-I) on 24-h GH release. Unless otherwise stated, all data are expressed as mean ± SEM. The level of statistical significance was set at *P* < 0.05. 

## 3. Results

### 3.1. Weight-Loss Study

At the conclusion of the weight-loss program, the 8 obese women that completed the weight loss program had lost approximately 30% of their initial body weight (30 ± 5 kg). The remaining 8 obese women dropped out during the study due to lack of motivation.

### 3.2. Sweat Secretion

**Figure 1:**
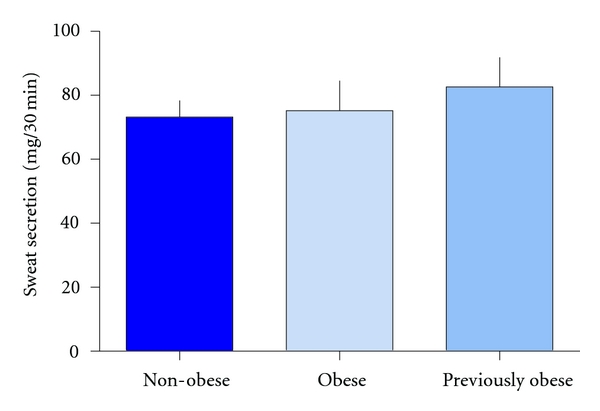
Sweat secretion rate. Columns represent (left to right) levels in non-obese (*n* = 16), obese (*n* = 16), and previously obese subjects (*n* = 8).

Sweat secretion was similar in obese and non-obese women (75 ± 9 versus 73 ± 5 mg/30 min), and no change in sweat secretion occurred after diet-induced weight loss (Figure 1).

### 3.3. 24-h GH Release and IGF-I Levels

**Figure 2:**
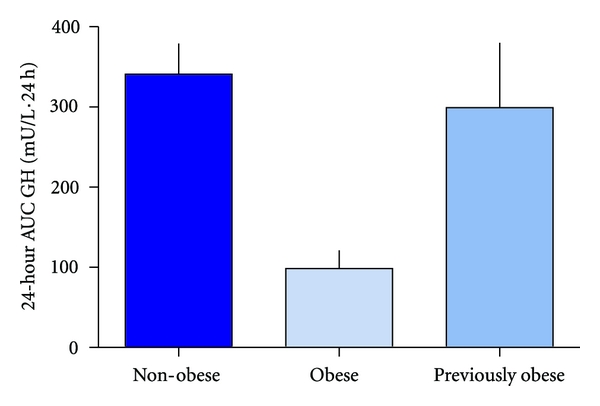
Twenty-four-hour GH release. Columns represent (left to right) levels in non-obese (*n* = 16), obese (*n* = 16), and previously obese subjects (*n* = 8).

Twenty-four-hour GH release was found to be reduced in obese women compared to non-obese women (Figure 2). A significant increase in GH levels was observed after weight loss, and no significant difference was present between previously obese and non-obese women (Figure 2). Basal levels of IGF-I were significantly lower in obese women at preweight loss compared to non-obese women and a significant increase was observed in IGF-I levels after weight loss, and no significant difference was present between previous obese and non-obese women (Table 1). Twenty-four-hour GH release and IGF-I data have previously been reported separately [23].

### 3.4. Insulin and FFA

FFA as well as insulin levels were found elevated (Table 1). No significant difference in FFA or insulin levels was present after weight loss between previously obese and non-obese women (Table 1).

### 3.5. Insulin Sensitivity

Insulin sensitivity estimated by the QUICKI formula was decreased in obese women compared to non-obese women. A significant increase in insulin sensitivity was observed after weight loss, and no significant difference was present between previously obese and non-obese women (Table 1).

### 3.6. Relationships between Insulin, Insulin Sensitivity, FFA, IGF-I, and 24-Hour GH Release

Regression analysis performed considering non-obese, obese, and previously obese subjects showed a significant positive correlation between insulin sensitivity and 24-hour GH release (Table 2).

## 4. Discussion

We have previously reported 24-h GH release and IGF-I levels to be impaired in obese subjects and normalised after diet-induced weight loss [23]. The novel data reported now imply that although GH deficiency and obesity in adults feature many similarities, we did not observe an impact on sweating capacity in the obese subjects despite markedly reduced spontaneous GH release and impaired circulating IGF-I levels. Thus, the subnormal sweat secretion rate in adults with GH deficiency reported in previous studies [1–4] as well as the positive correlations between circulating IGF-I levels and sweat secretion rate in these patients [1] may be specifically related to severe GH deficiency and not impaired GH-IGF-I axis *per se.* One explanation for the difference could be damage to the thermoregulatory centre in these patients with pituitary disease; however, parts of the GH-IGF axis seem to be present in human sweat glands [10, 24]. It could be speculated whether the normal sweat secretion observed in obese subjects with impaired GH-IGF-I axis adds further evidence that the criterion for the diagnosis of GHD in obese patients is complex. The mechanism behind the role of GH and IGF-I in sweat gland function remains speculative, and it cannot be ruled out that other factors than the GH-IGF-I axis in obese subjects are involved in the more profuse sweat secretion observed, although good correlation between whole body sweat secretion and regional sweat secretion rate has been reported [17]. 

A secondary objective of the study was to relate sweat secretion and GH-IGF-I to hyperinsulinaemia and elevated FFA often observed in obese subjects. Insulin and FFA are two of the main peripheral signals proposed to be interwined with the impaired GH release observed in obesity. We found no relationship between sweat secretion and insulin, or FFA levels. However, our data suggest that the decreased spontaneous 24-hour GH release observed in obesity seems related to the concomitant hyperinsulinaemia and insulin sensitivity rather than elevated FFA levels.

It has previously been reported that hyperinsulinaemia results in suppressed pituitary GH release [25–27]. In addition, circulating elevated FFAs have been suggested to be involved in the mechanism behind the hyposomatropism observed in obesity [28, 29]. In the present study, circulating FFA levels were significantly increased in obese subjects as reported by others; however, FFA levels were apparently not associated with the impaired physiological GH release. The improved GH response to stimulation tests in obese subjects pretreated with acipimox has been suggesting elevated FFA levels to be involved in the mechanism behind the GH hyposecretion observed in obesity [28, 29]. However, it is noteworthy that acipimox-induced decrease in FFA did not have an impact on physiological GH secretion [28]. Considering that acipimox is known to strongly reduce serum insulin in obese patients [30], it is speculated whether reductions in insulin levels *per se* might explain the magnitude of the acipimox-induced improvement in GH response. This can likely be explained as when insulin levels are significantly increased, insulin's role overcomes the effect of FFA on the GH release. 

In conclusion, we did not observe an impact on sweating capacity in the obese subjects despite markedly reduced spontaneous GH release and impaired circulating IGF-I levels.

## Figures and Tables

**Table 1:** Twenty-four-hour GH, basal levels of insulin, FFA, IGF-I levels, and insulin sensitivity in 16 obese, 8 previously obese, and 16 non-obese.

	Non-obese	Obese	Previously obese
24-hour AUC GH (mU/L·24 h)	341 ± 41	99 ± 23*	299 ± 81^‡^
IGF-I (*μ*g/L)	308 ± 18	220 ± 21*	330 ± 73^‡^
FFA (meq/L)	0.55 ± 0.07	0.80 ± 0.07**	0.63 ± 0.08^‡^
Insulin (pmol/L)	46.8 ± 3.6	81.9 ± 7.8*	43.3 ± 9.1^‡^
QUICKI (no units)	0.38 ± 0.01	0.33 ± 0.01*	0.39 ± 0.01^‡^

*P < 0.001 Obese versus non-obese.

**P < 0.05 Obese versus non-obese.

^‡^ *P* < 0.05 Obese versus previously obese.

**Table 2:** Correlations between 24-hour GH release, IGF-I, FFA insulin, and insulin sensitivity in all subjects (non-obese, obese, and previously obese subjects).

	24-hour GH release
Insulin	*r* = −0.59; *P* < 0.001
QUICKI	*r* = 0.72; *P* < 0.00001
FFA	*r* = −0.10; *ns*
IGF-I	*r* = 0.10; *ns*

## References

[1] Pedersen SA, Welling K, Michaelsen KF, Jorgensen JO, Christiansen JS, Skakkebaek NE (1989). Reduced sweating in adults with growth hormone deficiency. *Lancet*.

[2] Juul A, Skakkebaek NE (1991). Growth hormone deficiency and hyperthermia. *Lancet*.

[3] Sneppen SB, Main KM, Juul A (2000). Sweat secretion rates in growth hormone disorders. *Clinical Endocrinology*.

[4] Hasan W, Cowen T, Barnett PS, Elliot E, Coskeran P, Bouloux PMG (2001). The sweating apparatus in growth hormone deficiency, following treatment with r-hGH and in acromegaly. *Autonomic Neuroscience: Basic and Clinical*.

[5] Main K, Nilsson KO, Skakkebaek NE (1991). Influence of sex and growth hormone deficiency on sweating. *Scandinavian Journal of Clinical and Laboratory Investigation*.

[6] Main KM, Price DA, Savage MO, Skakkebae NE (1993). Decreased sweating in seven patients with Laron syndrome. *Journal of Clinical Endocrinology and Metabolism*.

[7] Sandahl Christiansen J, Jorgensen JO, Pedersen SA (1991). GH-Replacement therapy in adults. *Hormone Research*.

[8] Juul A, Behrenscheer A, Tims T, Nielsen B, Halkjaer-Kristensen J, Skakkebaek NE (1993). Impaired thermoregulation in adults with growth hormone deficiency during heat exposure and exercise. *Clinical Endocrinology*.

[9] Juul A, Hjortskov N, Jepsen LT (1995). Growth hormone deficiency and hyperthermia during exercise: a controlled study of sixteen GH-deficient patients. *Journal of Clinical Endocrinology and Metabolism*.

[10] Oakes SR, Haynes KM, Waters MJ, Herington AC, Werther GA (1992). Demonstration and localization of growth hormone receptor in human skin and skin fibroblasts. *Journal of Clinical Endocrinology and Metabolism*.

[11] Lange M, Thulesen J, Feldt-Rasmussen U (2001). Skin morphological changes in growth hormone deficiency and acromegaly. *European Journal of Endocrinology*.

[12] Carroll PV, Christ ER, Bengtsson BÅ (1998). Growth hormone deficiency in adulthood and the effects of growth hormone replacement: a review. *Journal of Clinical Endocrinology and Metabolism*.

[13] Rasmussen MH, Hvidberg A, Juul A (1995). Massive weight loss restores 24-hour growth hormone release profiles and serum insulin-like growth factor-I levels in obese subjects. *Journal of Clinical Endocrinology and Metabolism*.

[14] De Marinis L, Bianchi A, Mancini A (2004). Growth hormone secretion and leptin in morbid obesity before and after biliopancreatic diversion: relationships with insulin and body composition. *Journal of Clinical Endocrinology and Metabolism*.

[15] Savastano S, Di Somma C, Belfiore A (2006). Growth hormone status in morbidly obese subjects and correlation with body composition. *Journal of Endocrinological Investigation*.

[16] García Hidalgo L (2002). Dermatological complications of obesity. *American Journal of Clinical Dermatology*.

[17] Hjortskov N, Jepsen LT, Nielsen B, Juul A, Skakkebaek NE (1995). Pilocarpine iontophoresis test: an index of physiological sweat secretion?. *Clinical Physiology*.

[18] Juul A, Bang P, Hertel NT (1994). Serum insulin-like growth factor-I in 1030 healthy children, adolescents, and adults: relation to age, sex, stage of puberty, testicular size, and body mass index. *Journal of Clinical Endocrinology and Metabolism*.

[19] Andersen L, Dinesen B, Jorgensen PN, Poulsen F, Røder ME (1993). Enzyme immunoassay for intact human insulin in serum or plasma. *Clinical Chemistry*.

[20] Miles J, Glasscock R, Aikens J, Gerich J, Haymond M (1983). A microfluorometric method for the determination of free fatty acids in plasma. *Journal of Lipid Research*.

[21] Merriam GR, Wachter KW (1982). Algorithms for the study of episodic hormone secretion. *The American Journal of Physiology*.

[22] Katz A, Nambi SS, Mather K (2000). Quantitative insulin sensitivity check index: a simple, accurate method for assessing insulin sensitivity in humans. *Journal of Clinical Endocrinology and Metabolism*.

[23] Rasmussen MH, Juul A, Hilsted J (2007). Effect of weight loss on free insulin-like growth factor-I in obese women with hyposomatotropism. *Obesity*.

[24] Hodak E, Gottlieb AB, Anzilotti M, Krueger JG (1996). The insulin-like growth factor I receptor is expressed by epithelial cells with proliferative potential in human epidermis and skin appendages: correlation of increased expression with epidermal hyperplasia. *Journal of Investigative Dermatology*.

[25] Weaver JU, Noonan K, Kopelman PG (1991). An association between hypothalamic-pituitary dysfunction and peripheral endocrine function in extreme obesity. *Clinical Endocrinology*.

[26] Clasey JL, Weltman A, Patrie J (2001). Abdominal visceral fat and fasting insulin are important predictors of 24-hour GH release independent of age, gender, and other physiological factors. *Journal of Clinical Endocrinology and Metabolism*.

[27] Luque RM, Kineman RD (2006). Impact of obesity on the growth hormone axis: evidence for a direct inhibitory effect of hyperinsulinemia on pituitary function. *Endocrinology*.

[28] Cordido F, Peino R, Peñalva A, Alvarez CV, Casanueva FF, Dieguez C (1996). Impaired growth hormone secretion in obese subjects is partially reversed by acipimox-mediated plasma free fatty acid depression. *Journal of Clinical Endocrinology and Metabolism*.

[29] Pontiroli AE, Manzoni MF, Malighetti ME, Lanzi R (1996). Restoration of growth hormone (GH) response to GH-releasing hormone in elderly and obese subjects by acute pharmacological reduction of plasma free fatty acids. *Journal of Clinical Endocrinology and Metabolism*.

[30] Worm D, Henriksen JE, Vaag A, Thye-Rønn P, Melander A, Beck-Nielsen H (1994). Pronounced blood glucose-lowering effect of the antilipolytic drug acipimox in noninsulin-dependent diabetes mellitus patients during a 3-day intensified treatment period. *Journal of Clinical Endocrinology and Metabolism*.

